# Supplementary material for: Prevalence and transmission of the most relevant zoonotic and vector-borne pathogens in the Yucatan peninsula: A review
Source: PLoS Negl Trop Dis. 2024 Jul 3;18(7):e0012286. doi: 10.1371/journal.pntd.0012286 (PMC11251636; doi:10.1371/journal.pntd.0012286)
Supplement: Attachment — Submitted filename: Response to Editors & reviewers.pdf [file pntd.0012286.s002.pdf]

Response to Editors and reviewers

We thank the thoughtful constructive feedback of two reviewers, which helped us to improve the manuscript throughout.

We here include a 'Point-by-point response to reviewers', where we carefully answer every comment and suggestion made. To facilitate locating the changes we made, they are indicated with track changes in the revised version of the manuscript, and with blue lettering in the responses below in this document. Line numbers refer to those in the manuscript word document with track-changes.

Academic and Section Editors

Comments to the Author:

Thank you very much for submitting your manuscript "Prevalence and transmission of the most relevant zoonotic pathogens in the Yucatan peninsula: a review" for consideration at PLOS Neglected Tropical Diseases. As with all papers reviewed by the journal, your manuscript was reviewed by members of the editorial board and by several independent reviewers. In light of the reviews (below this email), we would like to invite the resubmission of a significantly-revised version that takes into account the reviewers' comments.

Reviewers found the topic of value but recommended significant restructuring, adding a multitude of studies that should have been considered, revising the search strategy, and excluding non-zoonotic pathogens. Your revision should include a point-by-point response to reviewer comments.

The topic is of value but needs significant restructuring, as it does not include a multitude of studies which should have been considered. This is due to the search strategy. On the other hand non-zoonotic pathogens should be excluded.

Response: Thanks for the positive criticism and for considering the topic of our revision valuable. We also appreciate the suggestions to better address the search strategy increasing the number of studies focusing on pathogens with zoonotic risk. We include below a point-by-point response to the reviewers' comments, which helped restructuring the manuscript and improving its content.

Reviewer #1:

Authors have identified using the PRISMA criteria to select studies from 2015 to 2023 with the search terms which they describe which primarily involves the term "zoonosis" or "zoonotic" and the Yucatan. I am concerned with the overall selection of the studies used in this article and the methods of identifying which studies to evaluate. For example, a recent study: "Trypanosoma cruzi in Mexican Neotropical vectors and mammals: wildlife, livestock, pets, and human population" PMID: 38060864, is not included in the investigation but should be given what the authors are wanting to analyze.

The problem lies in the use of "zoonosis" or "zoonotic" and in pure, this refers to the infectious diseases which are spread directly from non-human host to a human. Often that term is not used in the title of a research article if not assessing directly for zoonotic transmission. I can only imagine if the authors included vector-borne in the selection criteria how many more studies would have been selected and thus this analysis is missing likely many studies. Also, it may be a better idea to include in the search terms the specific pathogen and Yucatan and other regions of interest. For example, Trypanosoma cruzi and Yucatan, Leishmania and Yucatan...

Response: We thank the reviewer for highlighting some problems pertaining to our search strategy that led to missing some key studies.

As suggested by the reviewer, we performed a new search including an extended set of terms: “Trypanosoma cruzi”, “Leishmania”, “Toxoplasma gondii”, “Leptospira”, “Rickettsia”, “Dengue virus”, “Zyka virus”, “Chikungunya virus”, “arboviruses”, and “vector-borne diseases”. As we focus strictly on

the region of interest, the terms “Campeche”, “Quintana Roo”, “Yucatan”, and “Yucatan peninsula” were also included. The search was set to look for the selected terms in the title, in the keywords and in the abstract of the published literature (not just the title).

In this revised version we explain this complete search protocol in lines 136-170. It is important to note that we kept our specific objective and aims of our study, namely to evaluate the most recent decade on zoonotic diseases (2015-2024) for exclusively the Yucatán peninsula (Campeche, Yucatán and Quintana Roo states).

Reviewer #2:
Although the methodology presented accords with the article's objective, I have concerns about two aspects of it: 1. the control of sample bias resulting from the literature review. Although a weighted presence is provided, the amount of articles found still has an impact on it. It is recommended that the writers clarify this point better. 2. Since serological detection could affect results, is it possible that there will be more variety if only studies using molecular detection are taken into account when calculating prevalence?
Thank you for your observation. Following a new bibliographic search considering the reviewer’s #1 suggestion, the number of studies included in this revised version more than doubled, from 74 to 165 bibliographic sources. This expanded search, which incorporated an extended set of terms, aimed to minimize possible bias in the reported prevalence by significantly increasing the number of studies reviewed. The complete search protocol can be found in lines 136-170.

A total of 234 weighted prevalence (Pw) data were collected from molecular (186 studies) and serological (45 studies) techniques across various habitats, including urban, rural, ecotone, and sylvatic settings. Prevalences were analyzed to identify potential differences between habitats and pathogens, with the results presented in a new table (lines 170-185; Table 2).

The weighted prevalence calculation is supported by the authors citing Vigueras (2019); nonetheless, it is preferable to credit the original works where this calculation is proposed. You can review the following papers:
1.Garamszegi LZ, Møller AP. 2007 Prevalence of avian influenza and host ecology. Proc. Biol. Sci. R. Soc. 274, 2003–2012. (doi:10.1098/rspb.2007.0124)
2. Walther BA, Clayton DH, Cotgreave PC, Gregory RD, Price RD. 1995 Sampling effort and parasite species richness. Parasitol. Today 11, 306–310. (doi:10. 1016/0169-4758(95)80047-6)
Response: We appreciate the suggestion. We now include these original sources

Reviewer #3:
After reviewing the manuscript entitled "Prevalence and transmission of the most relevant zoonotic pathogens in the Yucatan peninsula: a review," here are the main comments and observations.
First, the manuscript is well written, including a relevant analysis of Zoonosis in the Yucatan peninsula in the last decade. The topic is pertinent because the region is experiencing rapid economic and urban development, significant environmental changes, and emerging diseases.
The methodological approach behind the review is sound, with proper documentation of the available bibliography.
Response: We thank the reviewer for the thoughtful comments.

Second, a significant issue for the manuscript is that some treated diseases are not zoonotic—specifically Blastocystis, Dengue, SIKA, and Chikungunya.
Response: Thank you for bringing this to our attention. Non-zoonotic pathogens were excluded from the analysis and the results section in this revised version.
When referring to Dengue, Zika, and Chikungunya, we consistently refer to them as mosquito-borne, vector-borne diseases (or similar) throughout the manuscript.

Third, the authors must include the proper analysis of the reviewed information on the ectoparasitic arthropods as zoonotic agents (not only vectors).
Response: Based on the data obtained with the extended bibliographic revision, we conducted a thorough analysis of the information on ectoparasitic arthropods, specifically focusing on infected species with vector-borne pathogens with zoonotic risks.

Fourth, the authors should revise the discussion of the findings because many of the paragraphs need more references, and some sections are speculative and not supported by the evidence provided in the revised manuscripts. Fifth, the conclusions must focus on the evidence-based findings extracted from the reviewed papers.
Response: We thoroughly revised the discussion and conclusions as indicated. This revised version has been changed/rephrased where appropriate to avoid speculation, as we describe in detail below for the different reviewer's specific comments.

Reviewer #1: Given the methods need attention I am not sure we have accurate results.
Response: We updated and improved the search protocol and analysis as suggested by reviewers; thus, we consider our results are fully supported in this revised version of the manuscript.

Reviewer #2: The reported results are clear and in accordance with the methodology that was provided. If there is any relationship between the cases in the bibliographic review and the cases reported in the epidemiology bulletin, it would be nice to know it.
Response: It would be interesting to explore such relation, however there were only eight revised bibliographies offering prevalence data on humans between 2015-2024, hindering a potential comparative analysis. Thus, we considered both sources of information complementary.

The results are categorized by state (Campeche, Yucatan, and Quintana Roo), however the introduction doesn't go into more depth on how the states differ from one another. It is suggested to be more precise so that readers unfamiliar with the studied geographic area would understand the variations identified by state.
Response: We appreciate the suggestion. We now include in the Introduction a brief description of the overall differences among the states (lines 86-94).

Reviewer #3: Great figures.
Figure 5. Please change the beetle silhouette for a bug and the domestic fly silhouette for a mosquito without a beak.
Response: Thanks! We changed the silhouettes in figure 5 as suggested

L277. "Relative high number". Please be accurate about quantities and comparisons.
Response: This sentence was deleted.

L280-281. Please provide the incidence values and comparison between them as evidence of the sentence.

Response: Done, the sentence now reads as: "Three pathogen groups were studied in all the habitats, *T. cruzi, Leptospira,* and *Leishmania* spp. Regarding host and collecting site, the highest *T. cruzi* prevalences occurred in dogs (mean±standard deviation, 71.32±78.65), opossums (63.30±66.79), pigs (56.64±73.35), humans (53.34±60.46), and triatomines *T. dimidiata* (67.62±32.11) from domestic and peridomestic environments. The highest *Leptospira* infections were found in wild crocodiles (88.85±24.08), dogs (58.6±43.77), bats (31.71±17.97), and rodents (18.18±12.42), while *Leishmania* mainly infected humans (45.33±58.16), rodents (37.47±29.74), and sand flies (22.74±26.27) present in rural settlements surrounded by local vegetation. The highest arbovirus incidence was detected in humans (93.17±89.02), mosquitoes (53.75±2676.87), and rodents (37.53±56.6), while for *Rickettsia* was in rodents (34.30±14.24), dogs (74.93±27.54), humans (25.85±31.76), and ticks (45.27±33.89) that fed on domestic and synanthropic animals (e.g. dogs, cats, horses, opossums, deers). Finally, dogs (192.10±2.55), cats (98.74±5.75), and pigs (109.45±58.65) distributed in rural sites had the highest prevalence of *T. gondii* (Table 3). (lines 465-488).

L295. Check the spelling of scientific names. Also, it should stand for homogeny in writing scientific names and abbreviations of the genera. A suggestion would be to write the full name the first time, including authority, and then use the abbreviation for the genera. Use the first two letters of the genera in those cases where a genera start with the same letter. Remember that some genera have their proper abbreviation nomenclature, like Phlebotominae (See Marcondes CB. A proposal of generic and subgeneric abbreviations for phlebotomine sandflies (Diptera: Psychodidae: Phlebotominae) of the world. Entomol News 2007;118(4): 351–356; doi: 10.3157/0013-872X(2007)118[351:APOGAS] 2.0.CO;2 and Marcondes CB. On the utilization of abbreviations for phlebotomine sand flies (Diptera: Psychodidae: Phlebotominae). J Med Entomol 2019;56(1):1; doi: 10.1093/jme/ tjy197).

Response: We appreciate the suggestion. In addition to those highlighted by the reviewer, scientific names were thoroughly revised and corrected where appropriate.

L311-312. Aedes taeniorhynchus and Ochlerotatus taeniorhynchus are the same. Ochlerotatus was degraded to a subgenus of Aedes. Please revise the nomenclatural updates.

Response: Corrected

L315. Revise the scientific names of the Leishmania parasites. Some names should be corrected.

Response: We revised and corrected the scientific names.

L348-349. There are two "finally". Please revise.

Response: Corrected.

L389. Revise the scientific names of the Leptospira. Some names should be corrected.

Response: We fully checked the Leptospira names for their correct spelling.

L414. Revise the use of the word "demonstrates." The tone in a review manuscript is important.

Response: We changed the word "demonstrates" with "highlights"

L444-448. Please add references to support the arguments.

Response: Gottdenker et al. 2014 and Esposito et al. 2023 references added

L486-487. Please add references to support the argument.

Response: Done (Touray et al. 2023)

L515-516. Please add the proper citation format.
Response: Done

L519-520. Revise the sentence for clearance.
Response: the sentence was changed for better understanding (lines 650-652) "... Furthermore, infections by pathogenic *Rickettsia* are also of concern due to their potential to affect different vectors and animal hosts and because of their impact on human health [40,45,46,104]."

L526. Do you mean companion animals?
Response: Right. We changed pets to companion animals.

L529-531. Revise the tone of the sentence because it is speculative. The review and the results do not support the arguments.
Response: We rephrased the sentence and added references supporting the statement (lines 663-665) "Evidence has shown that habitat modification by deforestation, urban expansion, and tourism, along with the effects of climate change, have an impact on the emergence and spread of zoonotic diseases (Rose et al. 2020; Casazza et al. 2021), which is a current scenario in the Yucatan peninsula."

L538. Please add references to support the argument.
Response: the reference was added (United Nations 2018 Report; https://esa.un.org/unpd/wup/)

L551-554. Revise the sentence. The argument is out of the context of the paragraph.
Response: The sentence was deleted, and the paragraph was modified for clarity: "The emergence of viral diseases has been linked to drastic changes in habitat and environmental conditions, both natural and caused by human activities. These conditions can facilitate the movement of pathogen hosts to areas inhabited by humans in search of shelter and food; areas that also lack their predators (i.e. biological controls) (Flores-Ferrer et al. 2023). In fact, arbovirus infections in the region are of great concern within the range of emerging diseases, so it is crucial to maintain monitoring programs to understand the patterns of viral diversity in fauna and the factors influencing the successful transmission between different species (Flores-Ferrer et al. 2023)." (lines 676-683)

L556-557. Please add references to support the argument.
Response: Done (Gottdenker et al. 2014, Esposito et al. 2023, Swei et al. 2020)

L560-563. Please revise the tone of the argument. If there were a lack of information, this review would not be possible. But there is no lack of information; more information and evidence of zoonotic diseases in the region are probably needed.
Response: We do not quite understand the comment. In the (original) lines indicated, the text said "Finally, the lack of investment in disease surveillance and care, inadequate healthcare services, lack of waste management, absence of prioritization guidelines from the health sector, and lack of knowledge among susceptible populations, exacerbate the problem (Hotez et al. 2015; Ibarra-Cerdeña et al. 2022).". We do not say that there is a lack of information (published in scientific sources, health reports, etc. in which indeed our review is based). The statement is in regards of healthcare, the knowledge among the general public.
For clarity, we modified the beginning, as "Finally, the low investment in disease surveillance and care, limited healthcare services, inadequate waste management, absence of prioritization guidelines from the

health sector, and limited knowledge among susceptible populations (due to deficient communication programs), exacerbate the problem [76,77].” (lines 690-694)

Reviewer #1: In my opinion, I think it is hard to make conclusions with what is being presented in this article.
Response: We updated and improved the search protocol as suggested by reviewers, gathered information from 165 articles and reports, and reanalyzed the data following a sound and comprehensive methodology. Hence, we consider our results and conclusions are fully supported in this revised version of the manuscript.

Reviewer #2: The conclusions are clear, and the work's weaknesses are highlighted, especially with regard to the publishing bias. The health implications and their relationship to epidemiological surveillance are highlighted.
Response: We truly appreciate the support expressed by the reviewer.

Reviewer #3: L567-571. Please revise the paragraph. The arguments are out of the context of the evidence gathered in the reviewed bibliography. Arguments are speculative for a conclusion.
L572-575. Again. The arguments in the paragraph are speculative since any documented viruses fall in the risk category that the authors describe. All described pathogens are endemic, but some are expanding their prevalence. However, particularly, the viruses considered in this review are already widely distributed in tropical regions of the world and have an endemic transmission state.
Response: The reviewer is right. We modified the first paragraph of the conclusions, being much more cautious and avoiding speculation.
It now reads: “Global trends in biodiversity loss, as well as the emergence and transmission of zoonotic diseases, have never been as relevant as they are nowadays. The COVID-19 pandemic has led to a crucial warning of the impact of zoonotic diseases on health, economy, and global stability, highlighting the need to understand zoonotic processes and risk factors. The pandemic underlined the importance of interdisciplinary protocols among different actors and institutions, as well as the need for further cooperation between academia and the health sector.” (lines 607-703)

L577. Please rephrase the paragraph. The use of the word "heterogeneus" is confusing.
Response: To express it clearer, we modified the sentence as: “The existing information on zoonotic diseases on the Yucatan peninsula is heterogeneous, namely some hosts, vectors, and transmission trends are well known but others are not; this also varies in the different states we assessed.” (lines 707-709)

L582-588. Please revise the paragraph. The arguments are speculative for the evidence from the reviewed bibliography, particularly for the review's conclusion.
Response: Given the trends of deforestation and habitat modification in the region of study, significantly based on economic and not biological/conservation grounds, and the direct and indirect consequences in terms of our study (zoonotic and vector-borne diseases), we consider our overall argument in this paragraph a crucial message.
We modified it to read (lines 709-726): “Therefore, it is imperative to reinforce practices aimed at increasing the knowledge, monitoring, prevention, and control of these diseases at the regional level. Safeguarding health, social, and economic security by building tools and programs that promote the preservation of the ecosystem's structure and processes on which we depend, fostering synergy among the government, research, industry, and health sectors, and with society, is an urgent challenge. The

conservation of natural ecosystems that solely depends on economic interests will have immeasurable and irreversible costs, impacting the less privileged populations. It is crucially urgent to take measures to protect the health of ecosystems, including controlling deforestation and protecting aquifers, with decision-making and planning instruments based on scientific knowledge. Finally, we emphasize the need of studies on a larger spatio-temporal scale, to better elucidate the interactions between pathogens, hosts, vectors, environment, sociocultural, and economic aspects in this and other tropical regions."

Reviewer #1: (No Response)

Reviewer #2: In lines 106 and 107 the authors give as examples of viruses Nipah and SARS among others and then state that these can be found in the geographical region studied. It is suggested to be careful and clarify this point.
Response: We appreciate the observation. We now indicate the dengue fever, Zika, rabies, West Nile virus, as viral agents that have been reported in the region (lines 119-121).

In line 392 the authors state that zoonoses are grouped into two groups; direct and indirect transmission. It is suggested to revise the classification because there can be vectorial transmission, direct transmission (Rabies) and indirect transmission.
Response: We added and explained the direct, indirect, and vectorial transmission as follows (lines 517-525).
Pathogens of zoonotic risk can be divided into three main groups: those that are transmitted by direct contact with animals (e.g. rabies virus); those transmitted indirectly through contact of mucous membranes and wounds with infected materials and ingestion of contaminated food and water (*Leptospira* spp., *T. gondii*, *Brucella* spp.); and those transmitted by vectors including arthropods (fleas, lice, ticks, mosquitoes) that can transmit bacteria, parasites, or viruses when they bite a host; for instance, West Nile Virus (transmitted by mosquitoes), Bubonic plague (transmitted by fleas), and *Rickettsia* (transmitted by ticks) [1, 74]. (Reyes-Novelo et al. 2011; Plowright et al. 2017).

In lines 444 - 449 the authors briefly explain the dilution effect, where they state that in conserved sites, the diversity of communities reduces the spread of zoonoses through the regulation of susceptible populations. However the degree to which biodiversity will regulate infection by a specific parasite is dependent on how deterministic host assembly is, whether the pathogen is niche- or dispersal-limited, and how increases in richness affect host and vector abundance. It is suggested that the concept be revised to provide a clearer explanation.
Response: We added information, broadening the explanation (lines 574-584): "This ecosystem service is known as the 'dilution effect of pathogens' (Keesing and Ostfeld 2021a; Civitello et al. 2022). The generality of the dilution effect hypothesis remains controversial, because multiple mechanisms can operate together in the same disease system (e.g. host quality, host and vector abundance), which challenges the empirical assessment of such systems (Kessing et al. 2006). On the other hand, metadata analyses have shown multiple evidence of dilution, independently of host density, study design, and type and specialization of parasites (Civitello et al. 2022); also, that some taxonomic groups that are more likely sources of zoonotic pathogens tend to thrive when biodiversity is lost (Kessing and Ostfeeld 2021b). For instance, rural areas host various susceptible hosts that, due to their proximity to and/or productive activities performed in the surrounding forests, have greater contact with the conserved habitat, including vectors and natural hosts of known and potential zoonotic pathogens (Hernández-Rivera et al. 2015)."

Reviewer #3: (No Response)

Reviewer #1: After reading the manuscript I continue to go back to how these studies were selected and it was not hard to find a study through my own search which should have been included in the analysis. Only having 74 studies seemed low as soon as I read it and confirmed once I started looking for studies that should have been included.
Here are some examples:
PMID: 37652628, PMID: 38060864, PMID: 36184108, PMID: 31593524...
Response: We appreciate the highlights and search recommendations from the reviewer. As indicated in the above answers, for this revised version we updated and improved the search protocol as suggested by reviewers, gathered information from 165 articles and reports, and reanalyzed the data following a sound and comprehensive methodology.
(the indicated references are included, except the PMID: 36184108 (DOI: 10.1016/j.vprsr.2022.100779); this study was done in the state of Queretaro, thus outside of our region of analysis)

Reviewer #2: It's an interesting study that offers a spotlight on the occurrence of pathogens and their relationship to land use change on the Yucatan Peninsula. It is obvious that one of the most common issues in this type of review is publication bias, but it is something that can be explained and justified. To have a better understanding of the results, a more detailed description of the differences between the region's states is recommended. It would have been interesting to debate or report on articles in which they searched for a pathogen other than the reported but found negative results.
Response: It is true that in any meta-analysis of revision of published literature there is always an inevitable bias; one can never capture the entire set of information but, on the other hand, it brings together as much of the information available as possible.
As suggested by the reviewer, we now include a more detailed description of the three states and their overall differences regarding landscape modification (lines 86-94).
About other pathogens than reported, we mentioned those with zoonotic risk.

Reviewer #3: L30-33. Please revise the sentence. It looks like a conclusion.
Response: We entirely modified the first lines of the abstract, it now reads: “Habitat modification and land use changes impact ecological interactions and alter the relationships between humans and nature. The Yucatan peninsula has experienced significant modification of the landscape at the local and regional scales, with negative effects on forest cover and biological diversity.” (lines 34-38)

L40. Revise the use of the word competent. If the authors consider that a competent zoonotic agent is correct, they should include the data and the analysis to prove parasite competence.
Response: The sentence was modified, so this no longer applies, as follows “*Trypanosoma cruzi* was the vector-borne agent with the largest number of infected vertebrate species in all habitats…” (lines 51-53)

L42-45. Please revise the sentence. The tone of the sentence is inappropriate for a revisión manuscript. Someone would use that sentence in an experimental or field study conclusion.
Response: We rephrased the sentence to indeed reflect what we found given the information revised. “Dogs, cats, backyard animals, and their hematophagous ectoparasites are the most likely species maintaining the transmission cycles in human settlements, while rodents, opossums, bats, and other synanthropic animals facilitate connection and transmission cycles between forested habitats with human-modified landscapes.” (lines 53-57)

L45-46. Again, a review manuscript should not conclude on the observations of the studies. Should conclude about tendencies or common results. The paragraph states that landscape structure and conditions favor the prevalence of different zoonotic diseases; however, it contradicts the data shown in the manuscript. The evidence showed that landscape structures display different prevalences between the diseases but are not meant to favor the prevalence.
Response: Thanks for pointing this out, we modified it as follows: "*Trypanosoma cruzi*, arbovirus, and *Leptospira* infections were the most prevalent in urban and rural settlements, whereas *Leishmania* and *Rickettsia* had similar prevalence across habitats, likely due to the diversity and abundance of the infected vectors involved." (lines 58-61)

L48-50. Please revise the sentence. The authors should estimate zoonotic disease incidence to support the argument.
Response: We rephrased the sentence as suggested: "Results suggest that prevalence of zoonotic and vector-borne diseases is higher in deforested areas and agricultural aggregates, and in sites with precarious health and infrastructure services." (lines 62-64)

L53-54. The socio-ecosystem perspective is interesting; however, the manuscript should use this approach to conclude it because the manuscript is "one health" oriented. Please provide the socio-ecosystemic framework in the manuscript to make sense of these arguments.
Response: Given that socio-ecosystem variables were not directly assessed in the current review, we decided to remove the term.

L63. Tropical Yucatan is redundant. Please correct.
Response: Considering that not all potential readers are familiar with the geographic location of the Yucatán peninsula, we chose to leave the tropical indication; in different published articles it has also been used.

L65-66. Revise the sentence. Too many "and."
Response: Done

L67-68. Revise the sentence. It is incongruent with sentences in the main text and the abstract regarding understanding the impact of zoonotic diseases. Some paragraphs answer the question of the impact.
Response: We changed the final sentence of the abstract: "Some hosts, vectors, and transmission trends of zoonotic and vector-borne diseases in the YP are well known but others remain poorly recognized. It is imperative to reinforce practices aimed at increasing the knowledge, monitoring, prevention, and control of these diseases at the regional level. We also emphasize the need to perform studies on a larger spatio-temporal scale under the socio-ecosystem perspective, to better elucidate the interactions between pathogens, hosts, vectors, environment, and sociocultural and economic aspects in this and many other tropical regions." (lines 70-76)
